# Anti-acid treatment for ulcerated early gastric cancer may be a treatment option avoiding unnecessary gastrectomy: a cohort study

**DOI:** 10.1097/MS9.0000000000000874

**Published:** 2023-06-05

**Authors:** Daisuke Suto, Masashi Yoshida, Takaaki Otake, Yosuke Osawa, Kiichi Sato, Hidehiko Yamada, Takayuki Akita, Hironori Ohdaira, Yutaka Suzuki, Yutaka Kohgo

**Affiliations:** a Department of Gastroenterology; b Department of Surgery, International University of Health and Welfare Hospital, 537-3, Iguchi, Nasushiobara, Tochigi, Japan

**Keywords:** endoscopic submucosal dissection, gastrectomy, pretreatment diagnosis, proton pump inhibitor, ulcerated early gastric cancer

## Abstract

Diagnosing the invasion depth of ulcerated early gastric cancer is usually inaccurate, especially for endoscopists in primary care clinics who are often not experts in this area. In reality, many patients with open ulcers who can be treated with endoscopic submucosal dissection (ESD) are referred for surgery.

**Materials and methods:**

Twelve patients with ulcerated early gastric cancer who were treated with proton pump inhibitors, including vonoprazan, and underwent ESD were included in the study. Conventional endoscopic and narrow-band images were evaluated by five board-certified endoscopists (two physicians: A, B, and three gastrointestinal surgeons: C, D, and E). They assessed the invasion depth, and the results were compared with the pathologic diagnosis.

**Results:**

The accuracy of the invasion depth diagnosis was 38.3%. According to the pretreatment diagnosis of invasion depth, gastrectomy was recommended for 41.7% (5/12) of the cases. However, histological examination revealed that additional gastrectomy was required in only one case (8.3%). Thus, in four out of five patients unnecessary gastrectomy could be avoided. Post-ESD mild melena occurred in only one case, and there was no case of perforation.

**Conclusion:**

Antiacid treatment contributed to avoid unnecessary gastrectomy in four out of five patients for whom gastrectomy was indicated based on an inaccurate pretreatment diagnosis of the invasion depth.

## Introduction

Highlights

- Evaluation of ulcerated gastric cancer invasion pretreatment diagnosis accuracy.
- Endoscopic submucosal dissection safety assessment after treating ulcers by proton pump inhibitors.
- Pretreatment diagnosis accuracy was low in patients with ulcerated gastric cancer.
- Endoscopic submucosal dissection after ulcer treatment can be a treatment option to avoid an unnecessary gastrectomy.

In order to decide on a treatment strategy against gastric cancers, an accurate preoperative evaluation of the depth of cancer invasion is essential. The accuracy of the depth diagnosis would help in deciding whether endoscopic submucosal dissection (ESD) can be performed or there is requirement for gastrectomy, to treat early gastric cancer. Accurate diagnosis of early gastric cancer relies on having a good grasp of the characteristics of early stage disease and learning effective methods of endoscopic observation. However, endoscopists who perform esophagogastroduodenoscopy are not always well-trained in diagnosing invasion depth. Thus, general endoscopists may refer a patient to a surgeon for surgical treatment if they detect early ulcerated stomach cancer during esophagogastroduodenoscopy. Oncologists often encounter cases in which the decision between ESD and gastrectomy is challenging, particularly in ulcerated early gastric cancer, though recent advances in endoscopic equipment and diagnostics have made it possible to accurately diagnose the invasion depth.

According to the Japanese gastric cancer treatment guidelines, ESD is indicated for the endoscopic diagnosis of intramucosal carcinoma (cT1a), differentiated carcinoma, and ulcer-positive lesions^1^. In other words, ESD may be possible if the ulcer is healed. Wang *et al*.^2^ as well as Myung *et al.* ^3^ reported a case series in which submucosal dissection was performed in patients with ulcerated early gastric cancer, and the ulcer healing rate was ~60 and 80%, respectively, when proton pump inhibitors (PPIs) were used for about 1 month^2^. In ~80% of healed ulcer cases, histological examination showed that the cancer was confined to the mucosa (T1a)^2^. In addition, the pretreatment diagnosis of invasion depth in ulcerated early gastric cancer is difficult, and the accuracy was 55–70%, although endoscopic ultrasonography was used^4–6^. In reports by Myung *et al.* and Wang and Shan, it is not specified how invasion depth was diagnosed before ESD^2,3^.

In this study, we examined pretreatment diagnosis by board-certified endoscopists, but nonspecialists in invasion depth diagnosis, to determine the feasibility of ESD after ulcer treatment. The article has been reported according to the Surgical CAse REport (SCARE) checklist^7^.

## Materials and methods

Between June 2014 and June 2020, 12 patients with ulcerated early gastric cancer treated with PPIs, including vonoprazan, who underwent ESD were included in the study. The study protocol was approved on 29 October 2022, by the Ethics Committee of the International University of Health and Welfare Hospita (approval number: 22-B-27) according to the principles outlined in the Declaration of Helsinki. We generally perform white light, narrow-band imaging, and ultrasound endoscopy of lesions in most cases before ESD treatment. Without being informed at all about the purpose of the research article, five endoscopists (A, B: fellows of the Japan Gastroenterological Endoscopy Society, C: gastrointestinal surgeon in Gastroenterology, D: gastrointestinal surgeon and trainer of the Japan Gastroenterological Endoscopy Society E: gastrointestinal surgeon and fellow of the Japan Gastroenterological Endoscopy Society), who perform routine endoscopic examinations, evaluated the invasion depth by two conventional endoscopic and narrow-band images. All of them are board-certified endoscopists but nonspecialists in diagnosing cancer invasion depth and do not perform ESD.

Patients with early gastric cancer and open ulcers received esomeprazole 20 mg, lansoprazole 30 mg, or vonoprazan 20 mg orally once daily from the day of esophagogastroduodenoscopy to the day of ESD for ~1 month. ESD was performed after ulcer healing was confirmed by esophagogastroduodenoscopy.

ESD was performed using a single-channel esophagogastroduodenoscopy (GIF Q260J; Olympus Corporation) and an electrosurgical generator (VIO 300D; ERBE Elektromedizin GmbH). A hyaluronic acid mixture containing indigo carmine was injected submucosally using a needle. Mucosal incision and submucosal dissection were performed using a dual knife (KD-650U; Olympus Corporation) after marking lesion margins. The work has been reported in line with the strengthening the reporting of cohort, cross-sectional and case-control studies in surgery (STROCSS) criteria^8^.

The study protocol was registered at the UMIN Clinical Trials Registry (https://upload.umin.ac.jp/cgi-open-bin/ctr_e/index.cgi), registration number UMIN000050786.

## Results

**Figure 1:**
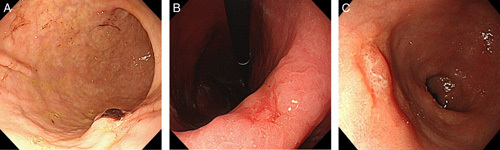
Representative images of pretreatment endoscopy. (A) Ulcerative gastric cancer with a clot attached to the posterior wall of the midbody. (B) An ulcerative gastric cancer lesion of approximately 10 mm on the lesser curvature of the angulus. (C) An ulcerative gastric cancer lesion of approximately 15 mm on the anterior wall of the antrum.

Patient characteristics and lesion sites are shown in (Table 1). Nine patients were male and three were female, with a mean age of 69.9 years (range 61–85 years). Six patients tested positive for *Helicobacter pylori* infection; they received no *Helicobacter pylori* eradication therapy before ESD. The highest number of lesions was in the middle region: one (8%), eight (67%), and three (25%) for the upper, middle, and lower regions, respectively, according to the Japanese classification of gastric carcinoma^8^. The overall accuracy for diagnosing invasion depth by the five endoscopists was 38.3%. The accuracy for diagnosing the invasion depth of ulcerated gastric cancer by the endoscopists was 7/12 (58.3%) for A and B, 5/12 (41.7%) for C, and 2/12 (16.7%) for D and E. In particular, the accuracy of the invasion depth by endoscopic imaging in Figure 1A–C was 0%.

**Table 1:** Patient characteristics

Characteristic	Men (*n*=9)	Women (*n*=3)	Total (*n*=12)
Age, years (mean)	61–80 (68.4)	67–85 (74.3)	61–85 (69.9)
Tumor location			
Upper	0	1	1
Middle	7	1	8
Lower	2	1	3
Tumor histopathology			
No tumor	0	0	0
Adenoma	0	1	1
Differentiated carcinoma	9	2	11
Undifferentiated carcinoma	0	0	0
Anticoagulants	0	1	1
Ulcer stage at first endoscopy			
Active	3	2	5
Healing	6	1	7
Macroscopic classification			
0-IIc: Superficial and depressed type	9	3	12
0-IIa: Superficial and elevated type	0	0	0
0-I: Protruded type	0	0	0
0-III: Excavated type	0	0	0
Pathological depth of invasion			
T1a (confined to the mucosa)	8	2	10
T1b (tumor invading the submucosa)	1	1	2
*Helicobacter pylori*infection			
positive	6	0	6
negative	3	3	6

The results of the ESD treatment are presented in (Table 2). According to the Japanese classification of gastric carcinoma^9,^ histological invasion depths were 83% (in 10 cases of mucosal cancer), 8.5% (in 1 case of submucosal 1 [SM1] invasive cancer), and 8.5% (in 1 case of SM2 invasive cancer). No intraoperative and postoperative perforations and hematemesis were observed. Postoperative second-look esophagogastroduodenoscopy revealed oozing in six cases (50%). Melena was observed in only one case after ESD. None of the patients required a blood transfusion.

**Table 2:** Patient treatment outcomes

Characteristic	Men (*n*=9)	Women (*n*=3)	Total (*n*=12)
Change of ulcer stage			
No change	0	0	0
Partial healing	2	0	2
Complete healing	7	3	10
Complete resection	9	3	12
Complications			
Delayed bleeding	0	0	0 (0%)
Hematemesis	0	0	0 (0%)
(Oozing): 2^nd^ look endoscopy	5	1	6 (50%)
Perforation	0	0	0 (0%)
Pathological ulceration			
UL(−): Ulcer or ulcer scar is absent	8	2	10 (83.3%)
UL(+): Ulcer or ulcer scar is present.	1	1	2 (16.7%)
Tumor size			
<10 mm	1	0	1
10–20 mm	4	2	6
20–30 mm	3	0	3
30 mm<	1	1	2
eCura classification			
eCuraA	7	3	10
eCuraB	1	0	1
eCuraC1	0	0	0
eCuraC-2	1	0	1

Histological diagnosis revealed 11 cases of differentiated carcinoma and one case of adenoma. The resection was classified as endoscopic curability A (eCuraA), eCuraB, and eCuraC-2 in 10, 1, and 1 cases, respectively, based on the Japanese guidelines for gastric cancer treatment. Endoscopic treatment was indicated for 11 patients, (91.7%), whereas one patient (8.3%) underwent additional gastrectomy. Thus, in four out of five patients unnecessary gastrectomy could be avoided.

## Discussion

ESD is often performed for early gastric cancer, but sometimes surgery is performed even in cases where ESD^10^ is indicated per the Japanese gastric cancer guidelines^1^.

In particular, many patients with ulcerated early gastric cancer who can be treated by ESD according to the Japanese gastric cancer guidelines are referred for surgery.

In the present study, the accuracy of diagnosing the invasion depth of ulcerated early gastric cancer was evaluated by five board-certified endoscopists who routinely perform esophagogastroduodenoscopy; the accuracy was low, at ~40%. The invasion depth diagnosis of ulcerated early gastric cancer tends to be deeper than the histological diagnosis. Even board-certified endoscopists often find it difficult to decide whether to perform ESD or surgical gastrectomy. This is the reason why many patients with ulcer are referred for surgery.

Previous reports have also reported a 55–70% accuracy for invasion depth diagnosis of ulcerated gastric cancer by endoscopic imaging and ultrasound endoscopy^4–6^. Therefore, the initial endoscopic image may be insufficient to determine the indication for surgery. In ulcerated gastric cancer, the gastric wall may be thickened by mucosal edema depending on the phase of the ulcer, which may lead to overestimation of the depth, while the depth may be underestimated when evaluated at the time of ulcer healing if apparent apical swelling or interruption is absent. Especially in an open ulcer, as was in case 12, the cancer invasion depth may be considered deep because of the edematous changes in the surrounding area.

The tumor (T)-stage positive diagnosis rate of endoscopic ultrasonography for ulcerated early gastric cancer is reported as 55–78% due to submucosal inflammation and fibrosis^4,5^. Re-evaluation of the tumor status with esophagogastroduodenoscopy after ulcer healing with PPIs use, including vonoprazan, is necessary in ulcerated early gastric cancer. An enormous number of endoscopic examinations are performed in private clinics. Endoscopists who find ulcerated early gastric cancer may refer patients to a surgery department requesting a gastrectomy, which can be a potential risk factor for unnecessary gastrectomy.

PPIs, including vonoprazan, mask early gastric cancer symptoms and may completely or partially improve endoscopic findings of malignant ulcers^11,12^. Ulcers in early gastric cancer have been treated with H2-receptor antagonists or PPIs^13,14^. In many ulcerated gastric cancer cases, gastrectomy can be avoided if the ulcer is treated with PPIs, including vonoprazan. Therefore, ESD after treatment of ulcers with PPIs may be a treatment option for ulcerated early gastric cancer.

ESD for ulcerated early gastric cancer increases the procedure time and the risk of adverse events such as perforation and bleeding during the procedure^15–17^. In the present study, ESD for early gastric cancer after healing ulceration was performed without the occurrence of perforation, and no patient required a blood transfusion. Post-ESD bleeding was reported in 6% of cases by Oda *et al.* and was significantly associated with location in the upper third of the stomach^18^. However, in the present study, procedure-related bleeding was not associated with clinicopathological features.

Complete resection was reported to be impossible in ESD for ulcerated early gastric cancer with a diameter of 21 mm and above^19^. Furthermore, a risk of noncurative resection in ESD for ulcerated gastric cancer has been reported when the lesion is located in the upper or middle part of the stomach^17^. In this study, 100% of patients underwent en-bloc resection.

The present study had some limitations. This study was conducted at a single institution and was a retrospective study using two types of images from a small number of cases. Therefore, large prospective observational studies are required to investigate these findings further.

In conclusion, antiacid treatment contributed to avoid unnecessary gastrectomy in four out of five patients who were for indicated gastrectomy based on an inaccurate pretreatment diagnosis of the invasion depth.

## Ethical approval

The study protocol was approved by the Ethics Committee of the International University of Health and Welfare Hospital (approval number: 22-B-27) according to the principles outlined in the Declaration of Helsinki.

## Consent

Participation was voluntary for all participants and all the participants had the right to withdraw at any time.

## Sources of funding

None.

## Author contribution

D.S. wrote the manuscript. M.Y., T.O., Y.O., K.S., H.Y., T.A., H.O., Y.S., and Y.K. reviewed the literature and contributed to manuscript drafting. Y.K. was responsible for the revision of the manuscript for important intellectual content. All authors approved the final version of the manuscript to be submitted.

## Conflict of interest

None.

## Research registration unique identifying number (UIN)

The study protocol was registered at UMIN Clinical Trials Registry (https://upload.umin.ac.jp/cgi-open-bin/ctr_e/index.cgi), registration number UMIN000050786.

## Guarantor

Daisuke Suto, First Author, Masashi Yoshida, Senior Author.

## Data availability statement

The datasets will be shared upon reasonable request.

## Provenance and peer review

Not commissioned, externally peer-reviewed.
